# Effectiveness of the Cooled Radiofrequency Ablation of Genicular Nerves in Patients with Chronic Knee Pain Due to Osteoarthritis: A Double-Blind, Randomized, Controlled Study

**DOI:** 10.3390/medicina60060857

**Published:** 2024-05-24

**Authors:** Hyun-Jung Kwon, Chan-Sik Kim, Doo-Hwan Kim, Jin-Woo Shin, Daeyun Choi, Seong-Soo Choi

**Affiliations:** Department of Anesthesiology and Pain Medicine, Asan Medical Center, University of Ulsan College of Medicine, 88, Olympic-ro 43-gil, Songpa-gu, Seoul 05505, Republic of Korea; kwonhj@amc.seoul.kr (H.-J.K.); chansik.kim86@gmail.com (C.-S.K.); dh_kim@amc.seoul.kr (D.-H.K.); sjinwoo@hotmail.com (J.-W.S.); iralux89@gmail.com (D.C.)

**Keywords:** arthralgia, chronic pain, denervation, knee, osteoarthritis, radiofrequency ablation, random allocation

## Abstract

*Background*: Increasing evidence supporting the clinical effectiveness of cooled radiofrequency ablation (RFA) therapy for genicular nerves in patients with chronic knee osteoarthritis (OA) exists. However, no study has been conducted to eliminate the potential influence of a placebo effect associated with this procedure. Therefore, we evaluated the efficacy of cooled RFA compared with a sham procedure in patients with painful knees due to OA. *Methods*: In this double-blind, randomized, controlled study, participants were randomly assigned to receive cooled RFA of the knee (cooled RFA group, n = 20) or a sham procedure (sham group, n = 20). The primary outcome was the proportion of successful responders at the three-month follow-up. The secondary outcomes were successful responders at one and six months; pain intensity of the knee; functional status; medication; and satisfaction at one, three, and six months after the procedures. *Results*: For the primary outcome, the successful responder rate was significantly higher in the cooled RFA group (76.5%) than in the sham group (33.3%) (*p* = 0.018). For the secondary outcome, more successful responders were observed in the cooled RFA group than in the sham group at one and six months after the procedure (*p* = 0.041 and 0.007, respectively). The decreased knee pain intensity was maintained throughout the six-month follow-up period in the cooled RFA group. No differences were observed in functional status, medication change, or satisfaction in both groups. *Conclusions*: The cooled RFA of genicular nerves offers significant pain relief and surpasses the effects attributable to a placebo.

## 1. Introduction

Knee osteoarthritis (OA) is a progressive degenerative disease of the knee joints and is commonly diagnosed in adults aged 50 or older [1]. The lifetime prevalence of knee OA is on the rise because of the aging population; therefore, it affects millions worldwide [2]. Moreover, knee OA is a leading cause of knee pain, which can significantly diminish an individual’s quality of life and can lead to physical activity reduction, disability, impaired sleep, and depression [1].

Conservative treatments are predominantly utilized to address knee pain due to OA before resorting to total knee arthroplasty. These conservative treatments encompass non-pharmacological approaches such as exercise programs and weight management and pharmacological options such as topical non-steroidal anti-inflammatory drugs, oral analgesics, and intra-articular injections with a steroid or hyaluronic acid [1,3]. Nevertheless, these measures are frequently insufficient for alleviating knee pain or cannot be used because of adverse effects [3].

Radiofrequency ablation (RFA) therapy for genicular nerves was introduced in 2010 and has proven effective as an alternative to conservative therapy [4,5]. Recently, the cooled RFA of genicular nerves, which is an advanced technique that uses a controlled cooling system, has been developed. The cooling mechanism is designed to minimize damage to adjacent structures and increase the size of lesions, which may enhance the safety and efficacy of the RFA treatment; these features have led to the growing application of cooled RFA on genicular neurotomy [6,7]. Furthermore, there is increasing evidence that supports the clinical efficacy of cooled RFA targeting genicular nerves for knee OA [8]. Nevertheless, no study has been conducted to eliminate the potential influence of a placebo effect associated with this procedure. Therefore, this study aimed to evaluate the efficacy of the cooled RFA of genicular nerves compared with the sham procedure in patients with chronic knee pain due to OA.

## 2. Materials and Methods

### 2.1. Study Design and Participants

This randomized, double-blind, sham-controlled study was conducted at the pain management clinic of our center. Approval to conduct this study was granted by our institutional review board (IRB approval number: 2020-0114), and written informed consent was obtained from each patient who participated in this study. All aspects of participant privacy and confidentiality were preserved. This study was registered with the Clinical Research Information Service (cris.nih.go.kr/KCT0004732) and was conducted following the Declaration of Helsinki. We followed the CONSORT guidelines in reporting this study.

### 2.2. Participants

Patients with unilateral chronic knee pain due to OA who visited the pain management clinic of our center between January 2023 and August 2023 were examined to ascertain their eligibility.

The inclusion criteria were as follows: degenerative knee OA of Kallgren–Lawrence grades II–IV confirmed using radiographic examination; persistent knee pain even after 6 months of conservative treatment such as physiotherapy, exercise therapy, or analgesics; the age of ≥50 or <80 years; pain intensity ≥ 6 (out of 10) on the numerical rating scale (NRS) (0 = no pain, 10 = unbearable pain); pain relief of at least 50% for more than 24 h after diagnostic genicular nerve block with local anesthetics under fluoroscopy or ultrasound guidance [4,9]; and patients who voluntarily agreed in writing to participate in this clinical trial.

The exclusion criteria were as follows: acute knee pain for less than six months; bilateral knee pain; prior knee surgery; other connective tissue disease affecting the knee; sciatica affecting the knee; hypersensitivity to local anesthetics or steroids; knee injection with steroids or hyaluronic acids within three months; coagulopathy, use of anticoagulants, or infection; and refusal to participate in this clinical trial.

### 2.3. Randomization and Blinding

Patients were randomly assigned to receive cooled RFA of the knee (cooled RFA group, n = 20) or genicular nerve block with a cooled radiofrequency (RF) cannula (sham group, n = 20) with a computer-generated randomization sequence. The randomization sequence was made using web-based randomization (http://www.randomizer.org, accessed on 22 October 2022) with a random block size of four and an allocation ratio of 1:1. The randomization sequence was concealed throughout the study from both the study participants and the outcome assessor, except for the physician who was responsible for pain treatment during the procedure.

### 2.4. Intervention

**Figure 1:**
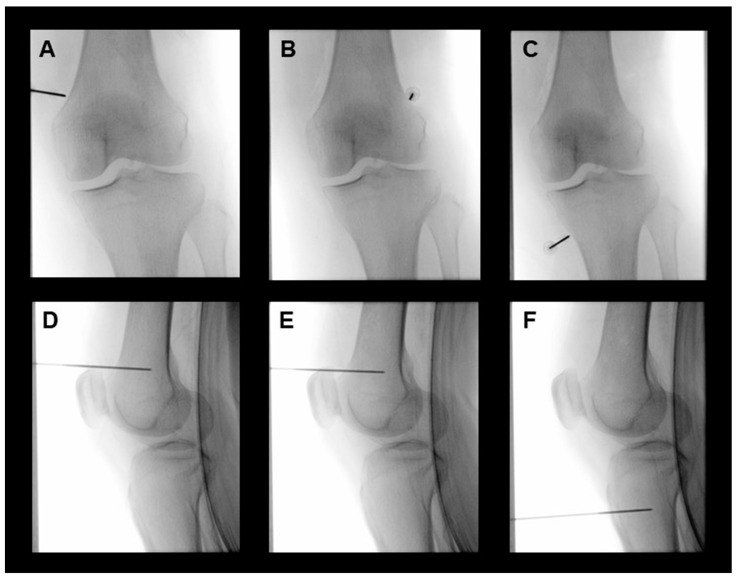
Representative fluoroscopic images of cooled RFA for the superomedial (**A**,**D**), superolateral (**B**,**E**), and inferomedial (**C**,**F**) genicular nerves of the left knee. Anteroposterior fluoroscopic view after cannula insertion for the placement of cooled radiofrequency electrodes into the junction between the shaft and epicondyle of the tibia and femur (**A**–**C**). Location of electrodes for the cooled RFA of genicular nerves in the lateral fluoroscopic image (**D**–**F**). RFA, radiofrequency ablation.

The patient was placed in a supine position on a fluoroscopy table, and a pillow was placed under the popliteal fossa of the affected knee to achieve a flexion of 10–15°. The patient was routinely monitored, and the procedure was performed under sterile conditions. The nerves targeted for treatment encompassed the superomedial, superolateral, and inferomedial genicular nerves in both groups, with the three target points following a standardized approach as outlined in the previously published literature [4]. The true anteroposterior fluoroscopic view of the tibiofemoral joint was obtained to show an open tibiofemoral joint space with equal-width interspaces on both sides. After identifying the target point, the skin and subcutaneous tissues were anesthetized with 2 mL of 2% lidocaine. The COOLIEF* Cooled Radiofrequency Kit (Avanos Medical Inc., Alpharetta, GA, USA), which has a 100 mm-long, 17-gauge straight RF introducer with a 4 mm active tip and an 18-gauge cooled RF probe with saline circuit, was employed for the technique. The cannula was advanced percutaneously toward the junction between the femoral or tibial shaft and the epicondyle under fluoroscopic guidance until contact was made with the bony cortex (Figure 1). Sensory stimulation < 0.6 V at 50 Hz was performed to identify the nerve position. A motor simulation of 2.0 V at 2 Hz was performed to test for the absence of fasciculation in the corresponding area of the lower extremity. Then, to provide anesthesia for the denervation, 2% lidocaine was injected through each introducer cannula. In the cooled RFA group, the cooled RF electrode was inserted through the cannula, and the RF generator (Baylis Medical Company, Inc., Montreal, QC, Canada) was activated. The electrode tip temperature was increased to 60 °C for 90 s. One RFA lesion was made for each genicular nerve. Sham participants underwent the same procedure without the activation of the RF generator, and the temperature of the electrode tip was not increased. A recorded mechanical sound of the RF generator was played for the same period. After the allocated procedure, 2 mL of 1% lidocaine and 5 mg of triamcinolone were injected into each treated site. Following the procedure, all patients were instructed to maintain their current medications for knee OA; minimal modifications to their medications and physiotherapy were allowed during the follow-up periods. The procedures were conducted by four pain physicians, and the patients remained unaware of the type of treatment administered.

### 2.5. Outcome Measures and Data Collection

The baseline characteristics, including age, sex, height, weight, body mass index, and coexisting medical conditions such as hypertension, diabetes, and depression, were assessed using the Patient Health Questionnaire-9 and recorded. Additionally, data on the target knee side were collected.

**Figure 2:**
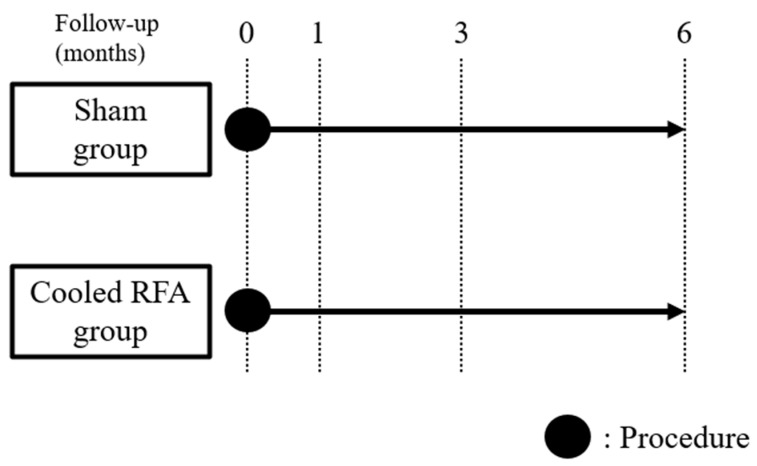
Study timeline diagram. Outcome assessments were conducted at baseline and one, three, and six months following the procedure. RFA, radiofrequency ablation.

The blinded assessor conducted outcome assessments at baseline and one, three, and six months following the procedure (Figure 2). The pain intensity of the affected knee was measured by the NRS. Physical function was assessed using the Western Ontario and McMaster Universities Arthritis Index (WOMAC) [10]. The Medication Quantification Scale III (MQS) was used to measure and quantify analgesic usage [11]. The Global Perceived Effect of Satisfaction (GPES) was measured according to the seven-point Likert scale (one = worst ever, two = much worse, three = worse, four = not improved but not worse, five = improved, six = much improved, and seven = best ever) to assess patient satisfaction and improvement [12].

The primary outcome was the proportion of successful responders three months after the procedure. Successful responders were defined as participants who experienced at least a 50% or four-point reduction in NRS for pain in the affected knee three months after the procedure [13]. The secondary outcomes were the proportion of participants who achieved at least a 50% or four-point reduction in knee pain at one and six months and in the pain intensity of the knee (NRS), WOMAC, MQS, and GPES at one, three, and six months. Any adverse effects (e.g., abnormal proprioception, numbness, paresthesia, neuralgia, motor weakness, and burns) were recorded during the follow-up period. All outcomes were evaluated during each outpatient visit or telephone consultation.

### 2.6. Statistical Analysis

To calculate the sample size of the trial, a pilot study was conducted by assigning 10 participants per group. Successful response was defined as a ≥50% or four-point decrease in NRS at three months after the procedure. Results showed that 60% of patients exhibited a successful response, whereas only 10% showed a successful response in the sham group. Accordingly, each group required 17 participants with a desired power of 0.8 and a two-tailed alpha error of 0.05. Considering a dropout rate of 15%, a total of 40 participants were required (20 in the sham group and 20 in the cooled RFA group).

The continuous variables of the baseline and procedural characteristics were summarized as mean with standard deviation or median with interquartile range. Categorical variables were represented as frequency (percentage). Between-group comparisons were evaluated using the Student’s *t*-test or the Mann–Whitney U-test for continuous variables and the chi-square test or Fisher’s exact test for categorical variables. All statistical analyses were conducted in the modified intention-to-treat population, which comprised patients who received the allocated intervention. Outcome values were analyzed by repeated-measures analysis of variance (ANOVA) with a Bonferroni post hoc test. Statistical significance was set at *p* < 0.05. Data manipulation and analyses were performed using MedCalc version 15.8 (MedCalc Software Ltd., Ostend, Belgium) and IBM SPSS version 22 (IBM Corp., Armonk, NY, USA).

## 3. Results

### 3.1. Study Population

**Figure 3:**
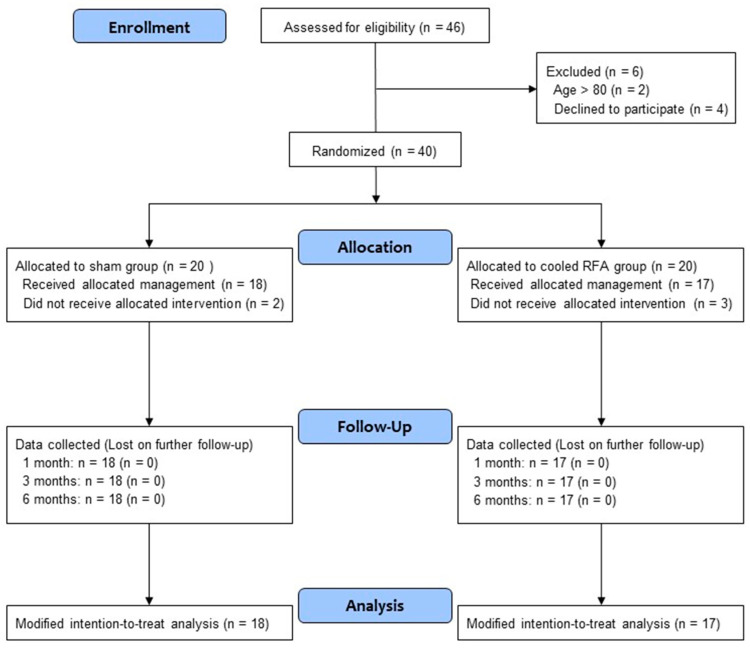
Study flow diagram. CONSORT flow diagram of patients included in the study. RFA, radiofrequency ablation.

Between January 2023 and August 2023, a total of 46 patients with chronic knee pain due to OA were screened for eligibility. Among them, 40 patients who satisfied the inclusion and exclusion criteria consented to participate in the trial. After randomization, 20 patients were allocated to each group (Figure 3). Five patients in both groups did not receive the allocated intervention because they did not visit again. Thus, a total of 35 patients (18 in the sham group and 17 in the cooled RFA group) were included in the modified intention-to-treat analysis. As shown in Table 1, the baseline characteristics were not significantly different between the two groups.

### 3.2. Primary and Secondary Outcomes

**Figure 4:**
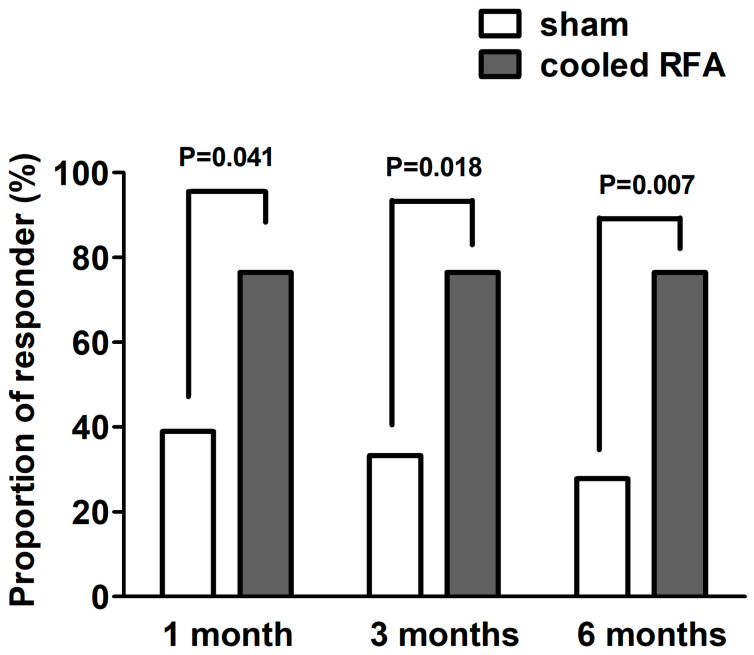
Proportions of successful responders one, three, and six months after sham or cooled RFA for genicular nerves in patients with knee osteoarthritis who do not respond to conservative treatment. A successful response was defined as a reduction of ≥50% from the baseline pain intensity or a reduction of ≥4 points from the baseline numerical rating scale for pain intensity. RFA, radiofrequency ablation.

For the primary outcome, 13 participants in the cooled RFA group (76.5%) obtained a successful response 3 months after the procedure, whereas 6 participants in the sham group (33.3%) obtained a successful response 3 months after the procedure (Table 2, Figure 4). The successful responder rate was significantly higher in the cooled RFA group than in the sham group (*p* = 0.018).

For the secondary outcome, the proportion of successful responders was significantly higher in the cooled RFA group than in the sham group at one and six months after the procedure (*p* = 0.041 and 0.007, respectively) (Table 2, Figure 3). Table 3 shows the results of the modified intention-to-treat analysis. The mean pain intensity (NRS) in the sham group was significantly improved only one month after the procedure compared with the baseline (*p* = 0.003). By contrast, the mean pain intensity (NRS) in the cooled RFA group was significantly improved at all post-procedure assessment points (one, three, and six months) compared with the baseline (*p* < 0.001). The group difference in pain intensity was not observed one and three months after the procedures, but the pain intensity was significantly lower in the cooled RFA group than in the sham group at six months (*p* = 0.041) (Table 3). Furthermore, the repeated-measures ANOVA revealed a significant interaction between time and group in terms of knee pain intensity (F = 4.5, df = 3.0, *p* = 0.005). As shown in Table 3, there were no significant improvements in WOMAC, MQS, and GPES at all post-procedure assessment points compared with the baseline, except WOMAC at one month (*p* = 0.047). No significant group differences were observed in WOMAC, MQS, and GPES at any period during the follow-up. In addition, there was no significant group-by-time interaction between the two groups over time in WOMAC, MQS, and GPES (Table 3). 

Any serious complications were not observed during the procedure. Although a few patients complained of transient pain during cannula insertion, none required additional medication or the discontinuation of the procedure. Furthermore, abnormal proprioception, numbness, paresthesia, neuralgia, or skin burns were not observed during the follow-up period. None of the patients complained of motor weakness.

## 4. Discussion

In patients with chronic knee pain due to OA, the proportion of patients with at least a 50% or four-point reduction in pain was significantly higher after the cooled RFA of genicular nerves than after the sham procedure for up to six months after the procedure. Furthermore, the decreased pain intensity of the affected knee was maintained throughout the six-month follow-up period in the cooled RFA group.

Since the first case report on the effectiveness of cooled RFA in patients with chronic knee pain [14], prospective and retrospective studies have been conducted to confirm the efficacy of the cooled RFA of genicular nerves in patients with chronic knee pain due to OA [8]. These studies have demonstrated that the cooled RFA of genicular nerves provides notable pain relief, improved functional outcomes, and positive patient-reported experiences in patients with chronic knee pain [8]. Moreover, recent prospective studies observed sustained significant pain relief for up to two years in approximately half of the patients who underwent this procedure [15,16]. Furthermore, there have been several comparative studies that demonstrated the superior effectiveness of cooled RFA to traditional treatments such as intra-articular steroid and hyaluronic acid injections and conventional RFA; however, further prospective studies are needed to corroborate these findings [3,17,18,19,20]. Meanwhile, no research has specifically addressed the placebo effect of this new technology. The possibility of a placebo effect should be considered because the power of expectations for innovative treatments is formidable and can significantly affect treatment results. [21] Accordingly, this study revealed that the cooled RFA of genicular nerves offers significant pain relief and surpasses the effects attributable to the placebo.

Conventional RFA operates by inducing thermal lesions within the targeted area, thus leading to the localized destruction of neuronal tissue and thermocoagulation. This disrupts the transmission of nociceptive pain signals from the peripheral nervous system to the central nervous system by trapping the targeted nerves within lesions [22]. Cooled RFA, which is an advancement in RFA technology, incorporates cooling mechanisms into the conventional technique. These cooling mechanisms involve circulating a cooling medium, such as saline, through electrodes to prevent the excessive heating of the surrounding tissue. Consequently, cooled RFA enables more precise and controlled applications of thermal energy [19]. Therefore, cooled RFA selectively disrupts the function of sensory nerve fibers without causing significant thermal damage to surrounding tissues [6,18,23,24]. Moreover, by maintaining a temperature-controlled environment throughout the ablation process [24], cooled RFA generates larger lesions than in conventional RFA, thus increasing the likelihood of intercepting aberrant pain pathways [6]. Due to the numerous neuroanatomical variations in the knee that make it difficult to accurately target nerves, cooled RFA may be more effective for pain relief by creating relatively large lesions [25]. Due to these mechanisms, the effectiveness and safety of the treatment could be optimized, thus making the cooled RFA of genicular nerves an effective and safe approach for relieving chronic knee pain due to OA.

This study has several limitations. First, this study has a relatively small sample size for observing potential adverse effects. Further research with a larger sample size and higher power would be beneficial for generalizing the study results. Second, the six-month follow-up period may be inadequate for assessing the long-term clinical effects of the cooled RFA of genicular nerves. Thus, all participants in this study are intended to be followed up for up to 12 months. Third, the protocol of the cooled RFA in this study did not incorporate the updated anatomical targets recommended in recent studies [25,26,27], but instead utilized traditional target sites. Furthermore, incorporating recent findings on anatomy may yield even better results. Lastly, higher mean 95% CI baseline NRS, WOMAC, and MQS scores in the cooled RFA group might potentially affect the outcomes, resulting in insignificant differences between the groups.

## 5. Conclusions

The cooled RFA of genicular nerves is regarded as an effective treatment option for chronic knee pain due to OA that does not respond to conservative treatment. The efficacy of this method cannot solely be attributed to the placebo effect and helps improve RFA treatment outcomes for chronic knee pain. Further prospective studies with longer follow-up periods are needed to better understand its efficacy and long-term effects, which may ascertain the usefulness of this new advanced treatment.

## Figures and Tables

**Table 1:** Baseline characteristics of the study participants.

Variables	Sham (n = 20)	Cooled RFA (n = 20)	*p* Value
Age (years)	71.9 ± 6.0	69.1 ± 11.8	0.354
Sex			0.716
Male	4 (20%)	6 (30%)	
Female	16 (80%)	14 (70%)	
Height (cm)	157.2 ± 6.5	156.7 ± 7.6	0.824
Weight (kg)	66.2 ± 11.3	62.4 ± 8.6	0.243
BMI (kg/m^2^)	26.7 ± 4.0	25.4 ± 3.3	0.271
Hypertension	8 (40%)	6 (30%)	0.741
Diabetes	4 (20%)	7 (35%)	0.480
PHQ-9	3.5 (1.5–13.5)	3.5 (1.5–10.5)	0.883
Target knee			0.205
Right	7 (35%)	12 (60%)	
Left	13 (65%)	8 (40%)	
Pain intensity (NRS)	6.3 ± 1.6	6.8 ± 1.6	0.283
WOMAC	56.2 ± 20.4	55.6 ± 21.1	0.922
MQS	4.6 (0.0–8.0)	3.9 (0.0–7.6)	0.638

Values are represented as mean ± SD, median (interquartile range), or number (%). BMI, body mass index; MQS, Medication Quantification Scale III; NRS, numerical rating scale of pain intensity; PHQ-9, Patient Health Questionnaire-9; RFA, radiofrequency ablation; WOMAC, Western Ontario and McMaster Universities Arthritis Index.

**Table 2:** Proportion of successful responders in the cooled RFA and sham groups in patients with knee OA who do not respond to conservative treatment.

Responder *	Sham (n = 18)	Cooled RFA (n = 17)	*p* Value
1 month	7 (38.9%)	13 (76.5%)	0.041
3 months	6 (33.3%)	13 (76.5%)	0.018
6 months	5 (27.8%)	13 (76.5%)	0.007

* Successful response was defined as ≥50% or a four-point reduction in the numerical rating scale of knee pain intensity. RFA, radiofrequency ablation; OA, osteoarthritis.

**Table 3:** Changes in the means of pain scores, physical function, Medication Quantification Scale, and GPES in the cooled RFA and sham groups in patients with knee OA who do not respond to conservative treatment.

Variables	Time	Mean (95% CI)		Difference (95% CI)	*p* Value
		Sham	Cooled RFA		
Pain (NRS)	Baseline	6.3 (5.6–7.1)	6.9 (6.0–7.8)	−0.5 (−1.7 to 0.6)	0.327
	1 month	3.9 (2.8–5.1) **	3.7 (2.8–4.6) ***	0.2 (−1.2 to 1.6)	0.733
	3 months	4.8 (3.7–5.9)	3.5 (2.7–4.4) ***	1.2 (−0.1 to 2.6)	0.063
	6 months	4.8 (3.8–5.8)	3.5 (2.6–4.4) ***	1.4 (0.1 to 2.7)	0.041
WOMAC	Baseline	55.3 (45.2–65.4)	59.0 (49.0–69.0)	−3.7 (−17.4 to 9.9)	0.583
	1 month	37.6 (26.9–48.3) *	45.4 (35.1–55.7)	−7.9 (−22.2 to 6.5)	0.273
	3 months	45.4 (36.3–54.5)	47.6 (39.0–56.3)	−2.3 (−14.4 to 9.9)	0.707
	6 months	41.9 (31.8–52.1)	49.1 (38.4–59.8)	−7.2 (−21.3 to 7.0)	0.311
MQS	Baseline	4.7 (2.4–7.0)	5.8 (2.0–9.6)	−1.1 (−5.3 to 3.1)	0.599
	1 month	5.0 (2.6–7.4)	5.3 (1.8–8.8)	−0.3 (−4.3 to 3.7)	0.889
	3 months	5.1 (2.8–7.3)	4.3 (1.0–7.5)	0.8 (−3.0 to 4.6)	0.666
	6 months	5.1 (2.8–7.3)	3.7 (0.8–6.6)	1.4 (−2.2 to 4.9)	0.431
GPES	1 month	5.1 (4.4–5.7)	5.0 (4.2–5.9)	0.1 (−1.0 to 1.1)	0.914
	3 months	5.0 (4.3–5.7)	5.4 (4.8–5.9)	−0.4 (−1.2 to 0.5)	0.428
	6 months	5.1 (4.3–5.9)	4.9 (4.1–5.7)	0.2 (−0.9 to 1.3)	0.677

Values are represented as the mean (95% CI) or mean difference (95% CI). NRS was used to assess the intensity of knee pain. WOMAC was used to assess physical function. MQS was used to quantify changes in analgesics. GPES was used to assess overall patient satisfaction. A repeated-measures ANOVA was used for the statistical analysis. * *p* < 0.05, ** *p* < 0.01, and *** *p* < 0.001 vs. baseline in each group, respectively. The *p* values for interactions between groups and time for knee pain, WOMAC, MQS, and GPES are 0.005, 0.744, 0.382, and 0.276, respectively. CI, confidence interval; GPES, Global Perceived Effect of Satisfaction; MQS, Medication Quantification Scale III; NRS, numerical rating scale of pain intensity; OA, osteoarthritis; RFA, radiofrequency ablation; WOMAC, Western Ontario and McMaster Universities Arthritis Index.

## Data Availability

The data presented in this study are available on request from the corresponding author due to privacy.
